# Developing the atlas of cancer in Queensland: methodological issues

**DOI:** 10.1186/1476-072X-10-9

**Published:** 2011-01-24

**Authors:** Susanna M Cramb, Kerrie L Mengersen, Peter D Baade

**Affiliations:** 1 Viertel Centre for Research in Cancer Control, Cancer Council Queensland, Gregory Tce, Fortitude Valley, Australia; 2 Centre for Data Analysis, Modelling and Computation, Queensland University of Technology, George St, Brisbane, Australia; 3 School of Public Health, Queensland University of Technology, Herston Rd, Kelvin Grove, Australia

## Abstract

**Background:**

Achieving health equity has been identified as a major challenge, both internationally and within Australia. Inequalities in cancer outcomes are well documented, and must be quantified before they can be addressed. One method of portraying geographical variation in data uses maps. Recently we have produced thematic maps showing the geographical variation in cancer incidence and survival across Queensland, Australia. This article documents the decisions and rationale used in producing these maps, with the aim to assist others in producing chronic disease atlases.

**Methods:**

Bayesian hierarchical models were used to produce the estimates. Justification for the cancers chosen, geographical areas used, modelling method, outcome measures mapped, production of the adjacency matrix, assessment of convergence, sensitivity analyses performed and determination of significant geographical variation is provided.

**Conclusions:**

Although careful consideration of many issues is required, chronic disease atlases are a useful tool for assessing and quantifying geographical inequalities. In addition they help focus research efforts to investigate why the observed inequalities exist, which in turn inform advocacy, policy, support and education programs designed to reduce these inequalities.

## Background

Since the 1978 declaration of Alma-Ata which highlighted the need to address inequalities in health status [1], there have been important advancements for cancer outcomes. Many developed nations have seen improvements in cancer survival, notably for colorectal cancer, breast cancer, prostate cancer, non-Hodgkin lymphoma and leukaemia [2,3]. Also, incidence and mortality rates for some cancers have declined [4]. However, notable inequalities in these outcomes persist, with numerous international studies reporting disparities in cancer outcomes across socioeconomic status or urban/rural categories [5-7].

Within Australia, one of the greatest recognised health challenges is achieving health equity for all [8]. Cancer patients living in rural and disadvantaged areas are generally more likely to be diagnosed with advanced cancer and have poorer survival outcomes [9,10]. Often these areas have a higher prevalence of risk factors such as smoking, obesity and lower levels of physical activity [11,12]. Distance is also important, with cancer patients in rural areas having reduced access to cancer care services [13-15].

Inequalities need to be quantified before they can be addressed. Maps have been used to portray geographical data for a range of diseases since the mid-1800s, including cancer [16]. By providing a visual representation of cancer outcomes, geographic patterns of disease are able to be identified and effectively addressed [17]. For example, cancer mortality maps showed high mortality from oral cancer in south-eastern United States of America which led to the identification of snuff dipping as a risk factor [18]. Similarly, mammography screening efforts were intensified after finding low in-situ breast cancer incidence rates from mapped data in north-eastern Connecticut [19].

We recently developed thematic maps showing the geographical variation in cancer incidence and survival across Queensland, Australia [20]. With a population of 4.2 million [21] and covering an area of 1.9 million square kilometres, Queensland has the country's most decentralized population [22] and the highest incidence of cancer [23]. As there is increasing interest in producing disease maps [24-30], it is hoped that by documenting the processes and rationale behind the many decisions made during the development of this Cancer Atlas, it may assist others seeking to produce similar types of chronic disease atlases.

## Methods

Ethical approval to conduct this study was obtained from the Queensland Health - Central Office Committee Human Research Ethics Committee (HREC/09/QHC/25). Approval to extract the data was obtained from the Chief Executive Officer - Centre for Health Care Improvement, Queensland Health, under delegation by the Director-General, Queensland Health.

### Data sources

The Queensland Cancer Registry (QCR) supplied de-identified data on all primary invasive cancers diagnosed among Queensland residents during 1996 to 2007. The QCR is a population-based cancer registry that maintains a record of all cases of cancer diagnosed in Queensland since 1982, with data currently available to the end of 2007 [31]. Survival status of all cancer patients is obtained through routine linkage with the (Australian) National Death Index, enabling deaths of cancer patients who die interstate to be identified. Across all cancers, 91% of cancers registered by the Queensland Cancer Registry in 2007 were histologically verified and 1.9% were registered based on death certificate only (DCO) [31]. Cases with unknown age group (0.001% of all cancers) were excluded from the analyses.

Estimated resident population data grouped by age group (0-4, 5-9..., 80-84, 85+), sex, year and statistical local area (SLA) were obtained from the Australian Bureau of Statistics. To calculate the expected population mortality estimates, de-identified unit record mortality data for all causes of death for Queensland residents were also obtained from the Australian Bureau of Statistics (ABS) [32].

### Choice of cancers

The Cancer Atlas described spatial variation in the leading cancers diagnosed in Queensland during the study period (Table 1). These included the (Australian) National Health Priority Area cancers of colorectal cancer, lung cancer, breast cancer, cervical cancer, prostate cancer and non-Hodgkin's lymphoma. Although a priority cancer, variation in non-melanocytic skin cancer was not assessed, since it is not routinely reported by population-based cancer registries in Australia. When a cancer was not gender specific, results were calculated for each gender. The only exception to this was breast cancer which was reported for females only due to the very small number of breast cancers diagnosed among males.

**Table 1:** Cancers examined for geographic variation, Queensland, 1998-2007

Type of cancer	ICD-O3 code	Total number males diagnosed	Total number females diagnosed
All invasive cancers	C00-C80 (excluding C44 (M805 to 811))	105,053	82,470
Bladder cancer	C67	5,034	1,571
Brain cancer	C70, C71, C72	1,504	1,067
Breast cancer	C50	Not included	22,420
Cervical cancer	C53	Not applicable	1,639
Colorectal cancer	C18-C20 and C218	13,405	10,871
Kidney cancer	C64-C66 and C68	3,117	1,883
Leukaemia	M980-M994	3,084	2,094
Lung cancer	C33-C34	11,152	5,683
Melanoma	C44 and M872-M879	13,793	10,110
Myeloma	M973	1,192	913
Non-Hodgkin lymphoma	M959, M967-M971	3,547	2,889
Oesophageal cancer	C15	1,464	639
Ovarian cancer	C56	Not applicable	2,120
Pancreatic cancer	C25	1,940	1,706
Prostate cancer	C61	25,222	Not applicable
Stomach cancer	C16	2,193	1,070
Thyroid cancer	C73	765	2,221
Uterine cancer	C54	Not applicable	3,112

### Geographical areas

SLAs were used to define the geographical areas. These are part of the Australian Standard Geographic Classification (ASGC) used by the ABS [33] and are often based on the incorporated bodies of local governments who are responsible for service provision and infrastructure at the local and regional level.

The ABS adjusts the geographical boundaries of SLAs according to changes in the population composition over time. To ensure statistical analyses referred to the same geographical area for the entire study period, all SLAs were mapped to the boundaries used for the 2006 ASGC. The mapping process was conducted within the Queensland Cancer Registry, and matched the suburb and postcode at diagnosis to the 2006 National Localities Index [34]. There were 478 SLAs in Queensland in 2006 [33].

Estimates of incidence and survival were also examined by area-level socioeconomic status and rurality. Socioeconomic status was defined using the Socioeconomic Indexes for Areas (SEIFA) Index of Relative Socioeconomic Advantage and Disadvantage (IRSAD) compiled by the ABS [35]. Queensland SLAs were ranked from the most disadvantaged to the most advantaged and then divided into quintiles, based on a variety of data items such as the percentages of: people with high income, people unemployed, households paying cheap rental, households with no car and households with broadband internet connection. Rurality was defined using the ARIA+ (Accessibility/Remoteness Index for Australia plus) classification [36], which defines remoteness on the basis of five categories: major city, inner regional, outer regional, remote and very remote. 'Remote' and 'very remote' categories were combined together. The level of remoteness is determined by road-based distance to services.

### Methods to generate estimates

To produce a useful map on a small-area scale it is important to have estimates that are robust, or relatively insensitive to outliers, across small areas. If estimates are not robust, these outliers from areas which are often based on very small populations, are more likely to be disproportionately influential, and thus compromise the overall interpretation of the map.

Modelling or smoothing methods are commonly used to generate robust estimates for small geographical areas. As traditional regression models are unable to incorporate spatial correlation, approaches which enable hierarchical structure to be incorporated such as generalised linear mixed models may be used. These may be calculated using either Bayesian, multi-level, or likelihood-based models, however, Bayesian methods do not require the restrictive distributional assumptions in the other models (such as, for example, Gaussian random effects) [37]. Smoothing methods require no distributional assumptions and include interpolation methods, or non-parametric such as kernel regression, kriging and partition methods [38,39]. They are generally easier and faster to perform than modelling, but a comparison of various modelling and smoothing methods suggested Bayesian models performed better than the smoothing methods [40].

Bayesian models incorporate empirical Bayes and fully Bayes methods. In both types of Bayesian models, parameters are assigned probability distributions, usually based on plausible or expected values, and termed 'priors'. Fully Bayesian methods assign second stage priors to the variance controlling this distribution ('hyperparameters'). In contrast, empirical Bayes methods estimate the hyperparameter from the distribution of the data [41]. Therefore, empirical Bayes methods give satisfactory point estimates, but are unlikely to provide accurate estimates of the associated uncertainty [42].

Fully Bayesian models are becoming increasingly common in disease mapping [43]. Advantages of Bayesian models in comparison to other methods include the ease of drawing strength from neighbouring regions so estimates are more reliable and robust, as well as providing better quantification of the uncertainty surrounding the calculated estimates [41,44]. Also, Bayesian methods enable structuring of more complicated models, inferences and analyses [45]. Other cancer atlases which have used fully Bayesian methods include NSW (Australia) [46] and Limburg (Belgium) [47] (Table 2).

**Table 2:** Selected Cancer Atlases published from 1995 onwards

Region	Time period	Outcome	Statistic mapped	Smoothing method	N regions^a^	N cancers mapped^b^	Presentation method^c^
Canada [63]	1986-1990	Incidence	CIF	None	290	17 (M, F or P)	Ecumene

Europe [64]	~1981-1990	Incidence Mortality	DSR	Floating average of neighbouring rates for non-cities	Not stated	31 (M, F)	Isopleth

India [65]	2001-2002	Incidence	DSR	None	593	1 (M, F)	Areal

Limburg [66] (Belgium)	1996-1998	Incidence	SIR	Poisson-Gamma and CAR Bayesian models	44	5 (M, F)	Areal

Netherlands [67]	1989-2003	Incidence	DSR	Floating average of neighbouring rates for non-cities	458	11 (M, F)	Isopleth

New York [68] (USA)	Not stated	Incidence	DSR	None	62	12 (M, F)	Areal

New South Wales [46] (Australia)	1998-2002	Incidence Mortality	SIR, SMR	CAR Bayesian model	192	22 - inc (M, F) 12-mort (M, F)	Areal

Pennsylvania [69] (USA)	1994-2002	Incidence	DSR	None	67	2 (M, F, P)	Areal

Queensland [20] (Australia)	1998-2007	Incidence Survival	SIR, RER	Bayesian hierarchical models: BYM and relative survival	478	19 (M, F)	Areal

South Australia [70] (Australia)	1991-2000	Incidence Mortality	DSR	None	117	11 (P)	Ecumene

Spain [71]	1987-1995	Mortality	SIR	Non-parametric empirical Bayes estimation method	2218	4 (M, F) out of 14 maps	Areal

Sweden [72]	1971-1989	Incidence	DSR, CIF	None	286	37 (M, F)	Areal

UK [73]	2003-2005	Incidence Survival Mortality	DSR, RS	None	350	17 (M, F, P)	Areal

UK/Ireland [74]	1991-2000	Incidence Mortality	CIF or CMF	None	127	21 (M, F)	Areal

USA [75]	1950-1994	Mortality	DSR, CIF	None	3055	41 (M, F)	Areal

a. When multiple areas are available, as for some of the online Atlases, the number of regions is the number at the most detailed level.

b. M = males, F = females and P = persons.

c. Ecumene means only populated areas were coloured, Areal indicates that each individual region was coloured, and Isopleth means a continuous gradient was used.

BYM = Besag, York and Mollié

CAR = Conditional AutoRegressive

CIF/CMF = Comparative Incidence/Mortality Figure, and is the ratio of the DSR of the area to the DSR of the entire region or country

DSR = Directly age Standardised Rates

RER = Relative Excess Risk of death

RS = Relative Survival

SIR/SMR = indirectly Standardised Incidence/Mortality Ratio

### Outcome measures - what to map?

#### Incidence estimates

Incidence is defined as the number of new invasive cancer cases diagnosed within a given time period. When examining incidence in small areas, the traditionally used estimate is the SIR (indirectly Standardised Incidence Ratio). The SIR is an estimate of relative risk within each area which compares the observed counts against an expected number of counts, based on the population size.

However, limitations associated with the SIR estimates have been previously noted [38]. For example, large differences can be observed in the SIR estimates even with relatively small changes in incidence counts, and areas with no cases automatically receive an SIR of zero, regardless of the expected counts [40].

Modelling the SIR via spatial or Bayesian methods overcomes many of these problems by producing more reliable and robust estimates. Although there are many advantages to using a modelled SIR, they reflect the comparison of SLA-specific estimates against the Queensland average and not comparisons between SLA-specific estimates themselves. The latter interpretation may be biased if the SLAs have different population age structures and the outcome measure varies by age. For this reason the maps must be interpreted in terms of which areas are higher or lower than the Queensland average [48]. Alternative measures, such as the Comparative Incidence Figure (CIF, which is the ratio of the local to national (or whole region) directly standardized rates, i.e. rates weighted by age groups using an external population), have been proposed to overcome this issue, but these have their own disadvantages, including larger standard errors [49]. In light of these evaluations, the modelled SIR was adopted.

#### Survival estimates

Typically, cancer atlases have tended to report variations in cancer mortality, rather than cancer survival (Table 2). However spatial variations in cancer mortality reflect differences according to where people die, which may not be where they resided when diagnosed or treated. Mortality data are also prone to bias from death certificate inaccuracies in cause of death classification [50]. In contrast, mapping cancer survival, which is the percentage of patients who survive for a given time after diagnosis, estimates the variation in outcomes based on where people lived when diagnosed. Since treatment generally occurs shortly following diagnosis, this better reflects the potential impact of barriers to treatment and support services.

Survival after the diagnosis of cancer is the most important single measure for monitoring and evaluating the early diagnosis and treatment components of cancer control [51]. When examining cancer survival using population-based data, relative survival is often the preferred method as it provides an estimate of the net cancer survival without errors from cause of death misclassification, including difficulties in assigning cause of death when cancer was a contributing cause, but may not be completely responsible for the death [52,53].

Relative survival aims to measure deaths in excess of what would be expected, that is, the proportion of cancer patients alive x years after diagnosis in the hypothetical situation where the cancer in question is the only possible cause of death. Relative survival is modelled via an excess mortality model, which contrasts the mortality in the general population with the mortality of cancer patients. The difference is assumed to be due to cancer-related deaths ('excess mortality'). This model generates the excess hazard, also called relative excess risk (RER).

The median smoothed RER (i.e. exponential of the sum of the spatial and random heterogeneity components) was mapped. Similar to the interpretation of the SIR, the RER is a comparison against the State average, and comparison between areas is not recommended.

### Bayesian hierarchical models

#### Incidence

For incidence models the Besag, York and Mollié (BYM) model was used, as it has been shown to have desirable properties for disease mapping [43]. This model is specified as:

$\begin{array}{l} y_{i}\sim \text{Poisson}(e_{i}\theta_{i})\\ \log(\theta_{i})=\alpha \;+u_{i}+v_{i} \end{array}$

where *e_i _*is the expected number of cases for the *i*th SLA, *θ_i _*is the standardised incidence ratio, α is the overall level of relative risk, *u_i _*is the spatial component modelled with the conditional autoregressive (CAR) prior, and *v_i _*is the unstructured random effects (which has a normal distribution centered around zero). Input data were aggregated over 1998 to 2007. Since incidence is likely to differ by gender, estimates for males and females were generated separately.

Since this is a fully Bayesian model, priors were specified for α, *u_i _*and *v_i_*. The prior for α was given a vague normal distribution with mean 0 and variance of 1.0 × 10^10^. The prior distributions for *u_i _*and *v_i _*required sensitivity analyses, and are discussed below.

#### Relative survival

For relative survival, a recommended approach is to model excess mortality under a generalized linear model based on collapsed data using exact survival times and a Poisson assumption [52]. The basic version of this model was extended to include spatial and random effects, similar to Fairley et al [54].

$$\begin{array}{l} \;\;\;\;\;\;\;\;\;\;\;\;\;\;\;\;\;\;\;\;\;\;\;\;d_{kji}\sim \text{Poisson}(\mu_{kji})\\ \log(\mu_{kji}-d_{kji}^{*})=\log(y_{kji})+\alpha_{j}+\text{x}\beta_{k}+u_{i}+v_{i} \end{array}$$

Where *y_kji _*is person-time at risk in the *k*th age group, the *j*th follow up interval and the *i*th SLA, $d_{kji}^{*}$ is the expected number of deaths due to causes other than the cancer of interest, *α_j _*is the intercept (which varied by follow-up year), *β_k _*is the coefficient of the predictor variable vector × (representing the broad age groups), *v_i _*is the unstructured random effects (which has a normal distribution) and *u_i _*is the spatial component modelled with the CAR prior. Both *α *and *β *were given priors with normal distributions having mean 0 and variance 1.0 × 10^6^. The model was run separately for males and females. Broad age groups were included in the model to prevent bias due to differing age structures between SLAs.

All cases considered 'at risk' during 1998 to 2007 were included. Since the earliest year of data was 1996, this meant that any cases diagnosed from 1996 onwards which were alive with up to 5 years follow-up at some stage during 1998-2007 were included. Cases alive on the 31^st ^December 2007 were considered censored.

This model excluded persons aged 90 years or older at time of diagnosis, those whose diagnosis was based on death certificate or autopsy only, or those with a survival time of zero days or less. In total, this was 3.3% of the records from 1996-2007.

### Adjacency matrix

Since the Bayesian models incorporate information from neighbouring regions, it is necessary to specify the definition of which SLAs are considered neighbours. An adjacency matrix is generated to apply these definitions in the Bayesian model. Using the standard terminology for adjacency options, which follow the possible moves of chess pieces, we used the "Queen" definition, so that SLAs were considered to be neighbours if they shared a common border [55]. The adjacency matrix was calculated using the program GeoDa [56] using 1^st ^order queen adjacencies. Although it is possible to use higher-order weights than first-order (e.g. second-order weights will include neighbours of neighbours), this was not considered useful for this analysis due to the much denser neighbourhood matrix and, particularly in rural areas of the state, the very large distances between second-order neighbouring SLAs.

Due to the large number of island SLAs in Queensland, 18 regions originally had no neighbours. Since estimates will not be smoothed unless a region has neighbours the default neighbourhood matrix was adjusted to ensure all regions had at least one neighbour. Additional neighbours were incorporated by considering they could share a border even if separated by a river, or a sea. In particular, most of the Far North islands were grouped together, with some mainland areas also included to ensure enough strength was provided to generate meaningful estimates that were able to converge.

### Computation

Models were run using WinBUGS [57] interfaced with Stata [58] (using the wb commands written by John Thompson, University of Leicester [59]) with a burn-in period of 100,000 iterations (incidence models) and 250,000 iterations (survival models) followed by 100,000 iterations. To decrease the correlation between iterations a subsample of every tenth iteration was kept. Only one chain was run for each estimate.

### Assessing convergence

Convergence was assessed using visual examination of trace, density and autocorrelation plots, as well as the Geweke diagnostic [60]. Geweke diagnostics were calculated as the difference between the means for the first 1000 iterations (10%) that were kept and the final 5000 iterations (50%), divided by the asymptotic standard error of the difference. These were generated for the SIR or RER estimates for all 478 SLAs, and any estimate that had a Geweke estimate with a p-value of less than 0.01 was considered unlikely to have converged. To save disk space and processing time, trace and density plots were only generated for 5% (n = 24) of the SLAs, composed of SLAs of concern due to small numbers as well as a random selection.

### Sensitivity analyses

For these types of models, and particularly when data are sparse, it is vital to carefully consider the choice of prior and compare the effects of alternate priors. The priors used on the distribution for the variance of the spatial and random effects components may particularly influence the results.

There were three stages to conducting the sensitivity analyses. First, the literature was searched to determine what priors were being used in similar models. Many BYM models were found, however, there were few examples of Bayesian relative survival models containing spatial components. As there was no other source of information relevant to the study at hand on which to base informative priors, a range of non-informative priors were used for the relative survival models. Second, the performance of each prior was evaluated. Since the potential influence of the prior will be more pronounced for scarce data, Tables 3 and 4 show some of the comparative numbers for a less common cancer - oesophageal cancer in males. In addition to these, observed values were plotted against those predicted by the model and quantile-quantile plots were examined. Third, convergence was examined, as outlined earlier. Lack of convergence could indicate a poor model, or it may simply indicate a longer burn-in period is required. Monitoring the estimate over the entire number of iterations (including burn-in) would show whether it is likely convergence will eventually be reached. In tables 3 and 4 the proportion of SLAs for which the SIR or RER estimate did not converge after discarding 50,000 iterations is shown.

**Table 3:** Sensitivity analyses for oesophageal cancer incidence among males

	Prior 1	Prior 2	Prior 3	Prior 4	Prior 5	Prior 6
Distribution of SIR						
						
Mean	100.8	99.4	101.5	100.7	100.6	103.6
Standard deviation	10.2	30.8	16.3	14.5	13.5	23.2
Maximum	140.6	455.1	181.2	169.5	166.4	201.8
75% Quartile	107.2	113.1	111.7	110.2	109.4	109.8
Median	96.5	93.5	95.1	95.6	95.9	95.7
25% Quartile	93.3	78.7	89.4	89.9	90.7	90.2
Minimum	87.4	55.9	79.6	79.3	80.0	79.8
90% ratio^1^	1.3	2.3	1.6	1.5	1.5	1.6
						
pD^2^	34.112	138.047	51.305	53.828	53.709	54.098
DIC^3^	1652.57	1660.32	1650.62	1648.51	1651.02	1650.71
Spatial fraction^4^	0.56	0.44	0.63	0.48	0.52	0.57
Percent SLAs with Geweke <0.01 for SIR	41.0%	1.9%	3.3%	9.4%	10.3%	10.5%

Notes:

1. The 90% ratio is calculated as the 95^th ^percentile divided by the 5^th ^percentile of the smoothed SIR estimates.

2. pD represents the effective number of parameters in the model. Larger values indicate less smoothing of estimates.

3. DIC = Deviance Information Criterion. Smaller values (of at least 5 below) indicate a better model fit.

4. The spatial fraction estimates the relative contribution of spatial and unstructured heterogeneity, and is calculated as: $\text{Spatial}\;\text{fraction}=\frac{\theta_{marginal}^{2}}{\theta_{marginal}^{2}+\sigma^{2}}$

where $\theta_{marginal}^{2}$ = marginal spatial variance, *σ *^2^= marginal variability of the unstructured random effects between areas. A value close to 1 indicates the spatial heterogeneity dominates, whereas a value close to 0 indicates the unstructured heterogeneity dominates.

**Table 4:** Sensitivity analyses for oesophageal cancer survival among males

	Prior 1	Prior 2	Prior 3	Prior 4	Prior 5	Prior 6
Distribution of RER						
						
Mean	100.2	100.7	100.4	100.1	100.4	100.0
Standard deviation	6.5	11.5	8.2	3.8	9.3	0.3
Maximum	119.6	140.7	127.6	111.3	129.5	102.1
75% Quartile	105.3	105.0	105.7	102.6	106.3	100.2
Median	98.0	97.3	97.7	99.2	97.0	100.0
25% Quartile	95.2	92.6	94.7	97.2	93.7	99.8
Minimum	80.9	63.4	75.0	89.4	72.5	98.3
90% ratio^1^	1.2	1.4	1.3	1.1	1.3	1.0
						
pD^2^	23.988	36.021	33.105	18.663	30.524	18.218
DIC^3^	3690.23	3690.27	3691.24	3691.32	3690.07	3694.96
Spatial fraction^4^	0.62	0.87	0.51	0.48	0.80	0.00
Percent SLAs with Geweke <0.01 for RER	89.3%	9.8%	10.5%	19.5%	21.5%	63.0%

Notes:

1. The 90% ratio is calculated as the 95^th ^percentile divided by the 5^th ^percentile of the smoothed RER estimates.

2. pD represents the effective number of parameters in the model. Larger values indicate less smoothing of estimates.

3. DIC = Deviance Information Criterion. Smaller values (of at least 5 below) indicate a better model fit.

4. The spatial fraction estimates the relative contribution of spatial and unstructured heterogeneity, and is calculated as: $\text{Spatial}\;\text{fraction}=\frac{\theta_{marginal}^{2}}{\theta_{marginal}^{2}+\sigma^{2}}$

Where $\theta_{marginal}^{2}$ = marginal spatial variance, *σ *^2^= marginal variability of the unstructured random effects between areas. A value close to 1 indicates the spatial heterogeneity dominates, whereas a value close to 0 indicates the unstructured heterogeneity dominates.

For the sensitivity tests some of the less common cancers were examined (for incidence: oesophageal, brain, myeloma; for survival (based on number of deaths): oesophageal, thyroid), as well as a more common one (incidence: melanoma; survival: pancreatic).

For both the incidence and survival models, *u *represents the spatial component, while *v *represents the random component. These components were each given hyperprior distributions, as below:

$$\begin{array}{l} \;\;\;\;\;\;\;\;\;\;\;\;\;\;\;\nu_{i}\sim N(0\text{,}\tau_{\nu }^{2})\\ \left[u_{i}|u_{j},i\neq j,\tau_{u}^{2}]\sim N({\bar{\mu }}_{i},\tau_{i}^{2}\right) \end{array}$$

$$\begin{array}{l} \text{where}\;\;{\bar{\mu }}_{i}=\frac{1}{\Sigma_{j}\omega_{ij}}\Sigma_{j}\mu_{j}\omega_{ij}\\ \;\;\;\;\;\;\;\;\;\;\;\;\;\;\;\;\tau_{i}^{2}=\frac{\tau_{u}^{2}}{\Sigma_{j}\omega_{ij}} \end{array}$$

*ω_ij _*= 1 if SLAs *i, j *are adjacent (or 0 if they are not).

The τ values control the variability of *u *and *v*. As such, the distribution can be specified using τ or *σ*, which is the square root of the inverse of τ (variance = *σ*^2^).

The following priors were compared for the incidence model:

1. τ*_u _*~ Gamma(0.5, 0.0005), τ*_v _*~ Gamma(0.5, 0.0005)

2. τ*_u _*~ Gamma(1,1), τ*_v _*~ Gamma(7.801, 2.793)

3. τ*_u _*~ Gamma(0.1, 0.1), τ*_v _*~ Gamma(0.001, 0.001)

4. τ*_u _*~ Gamma(0.1, 0.01), τ*_v _*~ Gamma(0.1, 0.01)

5. Reparameterised on *σ*: *σ_u _*~ Uniform(0,1), *σ_v _*~ Uniform (0,1)

6. Reparameterised on *σ*: *σ_u _*~ Uniform (0,1000), *σ_v _*~ Uniform (0,1000)

Prior 1 shrunk the estimates more than any other (the pD value is lower than the others, and the standard deviation smaller) (Table 3). Prior 2 induced far less shrinkage than the others (higher pD and standard deviation). Prior 2 also had a larger DIC (greater than 6 above the others), indicating worse model fit. Priors 3 to 6 gave fairly similar results, although prior 6 had a larger standard deviation.

It was decided to use prior 3 as it provided consistently plausible results and converged well across the range of cancers examined.

For the survival model, the following non-informative priors were compared:

1. τ*_u _*~ Gamma(0.5, 0.001), τ*_v _*~ Gamma(0.5, 0.001)

2. τ*_u _*~ Gamma (0.1, 0.1), τ*_v _*~ Gamma(0.001, 0.001)

3. τ*_u _*~ Gamma(0.1, 0.01), τ*_v _*~ Gamma(0.1, 0.01)

4. τ*_u _*~ Gamma(0.5, 0.0005), τ*_v _*~ Gamma(0.5, 0.0005)

5. Reparameterised on *σ*: *σ_u _*~ Uniform(0,1), *σ_v _*~ Uniform(0,1)

6. Reparameterised on *σ*: *σ_u _*~ Uniform(0,1000), *σ_v _*~ Uniform(0,1000)

Prior 3 was chosen because it demonstrated greater convergence properties across the range of cancers examined, while restricting the results to a narrower range of smoothed estimates than Prior 2 (Table 4).

### Production of maps

**Figure 1:**
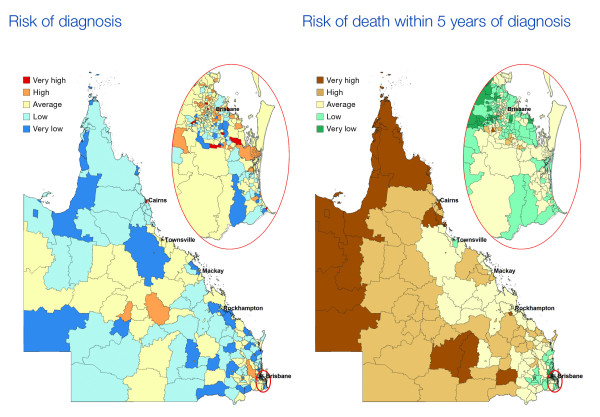
**An example of the incidence (risk of diagnosis) and survival (risk of death within 5 years of diagnosis) maps for all invasive cancers, males**.

A thematic scheme was chosen, with colours determined using color brewer http://colorbrewer2.org under the specifications of a diverging colour-scheme of 5 categories which are suitable for print and colour-blind friendly (Figure 1). The SIRs and RERs were categorised as 10% above and 30% above the State average, and the inverse of these for the lower cut-offs. There is great variability in the categories used in other atlases, but these fairly broad categories were used to reduce the probability of reporting spuriously significant differences.

Mapping alternative measures, such as the posterior probability of exceeding a certain value, were considered, but were deemed unsatisfactory due to difficulties in interpretation and the lack of information provided in regards to the size of the risk [27,44]. Therefore we used graphs to show the precision of the mapped estimates.

#### Graphs

**Figure 2:**
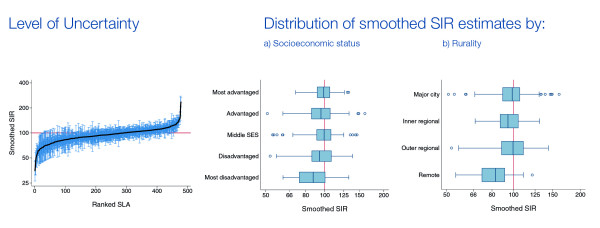
**An example of the incidence graphs for all invasive cancers, males**.

To supplement the information provided in the maps, a graph showing the ranked SIR or RER with the associated 95% credible interval for each SLA was provided (Figure 2). Horizontal box plots of the SIR or RER estimates by socioeconomic status and rurality were also provided to provide additional information about where the extent of variability across the state (Figure 2). Since a primary purpose of the model was to provide overall estimates of variability across the State, we did not include these additional variables in the model.

#### Additional data included

For each cancer with significant variation, SIR or RER estimates with 95% credible intervals were also provided by socioeconomic and rurality classifications. To calculate these, each iteration of the 10,000 iterations had the modelled observed value (incidence) and the adjusted deaths value (survival) calculated as above. For survival (which incorporated age group and time period) these were summed to give 10,000 iterations for each SLA. Each SLA was then grouped into rurality or socioeconomic status categories, and the adjusted estimates summed. These were divided by the original expected values to produce 10,000 SIR or RER estimates by rurality and socioeconomic status categories. The median of these 10,000 was used as the SIR or RER point estimate, and the 2.5 and 97.5 percentiles used to provide the lower and upper credible interval estimates, respectively.

### Determining whether observed variation is significant

Once the results have been produced and mapped, it is important to determine whether the apparent variation reflects true geographic differences. Therefore, a test for global clustering was conducted. Multiple tests are available [19], such as Besag-Newell's R, Moran's I, Oden's Ipop, but we elected to use Tango's MEET (Maximised Excess Events Test) [61] as it has been shown to perform well across a variety of datasets [19].

A small p-value from Tango's MEET indicates that estimates differ between regions. As is consistent with standard statistical analysis [62], adjusted p-values from the Tango's MEET statistic below 0.01 were considered to strongly indicate spatial variation, while values between 0.05 and 0.01 were moderately indicative of variation. Values of 0.05 or above were considered to not be significant, however two categories were defined. Values between 0.05 and 0.10 were considered to provide only weak evidence of geographic variation, while values above 0.10 no evidence of geographical variation.

Since Tango's MEET is calculated using Monte Carlo replications, it is expected that there could be slight variations in the results. To increase our confidence that the final classification of geographic variation was stable, Tango's MEET was run an additional 5 times for each cancer and gender combination. There were only two cases where the final classification did change for different replication, and so these cancers were assigned to the more conservative, less significant category.

Input for Tango's MEET requires an observed and expected value. Since the modelled results were of interest, the modelled observed value needed to be calculated. For the incidence data, the observed value was calculated by the smoothed SIR median value multiplied by the expected value to produce a modelled observed value. For the relative survival model, the adjusted deaths for each data point were calculated as: (person-time at risk × exp^follow-up time ^× exp^age group ^× RER) + expected number of deaths due to causes other than the cancer of interest.

i.e. $(y_{kji}\times {\text{e}}^{\alpha_{j}}\times {\text{e}}^{\beta_{k}}\times {\text{e}}^{u_{i}+\nu_{i}})+d_{kji}^{*}$

These were then added together for each SLA to provide the input data for Tango's MEET.

## Conclusions

Chronic disease atlases are a useful tool for assessing and quantifying geographical inequalities, as well as assisting to focus research efforts in investigating why the observed inequalities exist. When developing these atlases, a myriad of decisions concerning how to model and present the results need to be made and this paper presents one decision-making algorithm used to generate a cancer atlas.

There are several priority areas for future consideration in disease mapping including communicating spatial results, particularly finding ways to present the uncertainty surrounding the results; and the development and use of alternative statistical models such as classification and regression tree (CART) models. In addition, more detailed statistical models can be developed to investigate the impact of rurality, area-level and individual level socioeconomic status as well as temporal changes.

As with all chronic disease atlases, it is hoped that the presented variations in outcomes will stimulate further research efforts to investigate the reasons underlying the disparities and inform advocacy, policy, support and education programs to effectively address these, so that health equity will become a reality.

The full report is available (from February 2011) at: http://www.cancerqld.org.au/pdf/cancer_atlas.pdf

## Competing interests

The authors declare that they have no competing interests.

## Authors' contributions

PDB and KLM conceived the study. SMC performed the analysis. SMC and PDB drafted the manuscript. All authors contributed to, read and approved the final manuscript.
